# Novel insights into the roles of migrasome in cancer

**DOI:** 10.1007/s12672-024-00942-0

**Published:** 2024-05-15

**Authors:** Sijun Deng, Yiwen Wu, Sheng Huang, Xiaoyan Yang

**Affiliations:** School of Pharmaceutical Science, Hengyang Medical College, University of South China, 28 Western Changsheng Road, Hengyang , 421001 Hunan, People’s Republic of China

**Keywords:** Cell migration, Cancer, Migrasome, Specific marker proteins of migrasome, Migracytosis

## Abstract

Cell migration, a hallmark of cancer malignancy, plays a critical role in cancers. Improperly initiated or misdirected cell migration can lead to invasive metastatic cancer. Migrasomes are newly discovered vesicular cellular organelles produced by migrating cells and depending on cell migration. Four marker proteins [NDST1 (bifunctionalheparan sulfate N-deacetylase/N-sulfotransferase 1), EOGT (Epidermal growth factor domains pecific O-linked N-acetylglucosaminetransferase), CPQ (carboxypeptidase Q), and PIGK (phosphatidylinositol glycan anchor biosynthesis, class K)] of migrasomes were successfully identified. There are three marker proteins (NDST1, PIGK, and EOGT) of migrasome expressed in cancer. In this review, we will discuss the process of migrasome discovery, the formation of migrasome, the possible functions of migrasome, and the differences between migrasomes and exosomes, especially, the biological functions of migrasome marker proteins in cancer, and discuss some possible roles of migrasomes in cancer. We speculate that migrasomes and migracytosis can play key roles in regulating the development of cancer.

## Introduction

Cell migration, as an adaptive process[1], is crucial to normal physiological processes (development, immune defense, and wound healing) and pathology of multicellular animals[2]. It is also a hallmark of cancer malignancy[3]. Cancer metastasis contributes to plenty of patient deaths from solid tumors. Cell migration is a pivotal step in the metastatic process[4]. Cancer cells, migrating from tumor foci, flow into the bloodstream or lymphatic system, thereby leading to the form of distant metastatic tumor colonies[5]. Notably, inappropriate initiation or giving the wrong direction of cell migration may result in greatly enhanced tumor aggressiveness or metastasization[6]. Targeting the migration and spreading of cancer cells will be a promising adjunct to the treatment of patients with aggressive or locally invasive cancer.

Migrasomes are first described in 2015[7], which are formed at the tips or intersections of the RFs (retraction fibers). The RFs were first discovered and named by Taylor and Robbins in 1963[8]. They performed detailed light microscopy and TEM study, and observed the formation of long tubular structures, released by different types of migrating cells[8]. Little literature exists on the function of RFs, mainly about their relationship with the migrisome and Glioblastoma (GBM). Formation of RFs can coordinate the formation of migrasomes. In the study of Fan and colleague[9], they witness that cells form fewer migrasomes when making turns due to less derived RFs and the length of derived RFs controlled by the speed of cell migration would limit migrasome formation. Glioblastoma (GBM) is a refractory disease that has a highly infiltrative characteristic[10]. Over the past decade, GBM perivascular niche (PVN) has been described as a route of dissemination[11]. Trailed membrane structures, namely RFs, are formed by perivascular extracellular matrix (ECM) proteins. Lee et al.[12] validated that the ECM-related genes were highly expressed in the cells within the perivascular niche (PVN) where fibronectin (FN) induced RF formation and integrin α5β1 was identified as the main regulator of RF formation. They concluded that RFs produced by fibronectin-integrin α5β1 interaction can promote motility of brain tumor cells. From the above studies of RFs, migrasome formation depends on the ECM and fibronectin-integrin α5β1 interaction.

Notably, migrasomes perform a double and opposite function in (patho-)physiological contexts[13].

Since cell migration is crucial to cancer development, and the migrasome originates from cell migration, what is the relationship between migrasome and cancer development? Does migrasome have some biological functions? Studying the role of migrasome marker proteins in cancer is likely to reveal a mysterious relationship between migrasome and cancers. Targeting cancer cell migration-associated migrasome formation would be a promising adjunct in the treatment of aggressive cancer patients.

## The formation and contents of migrasomes

**Fig. 1:**
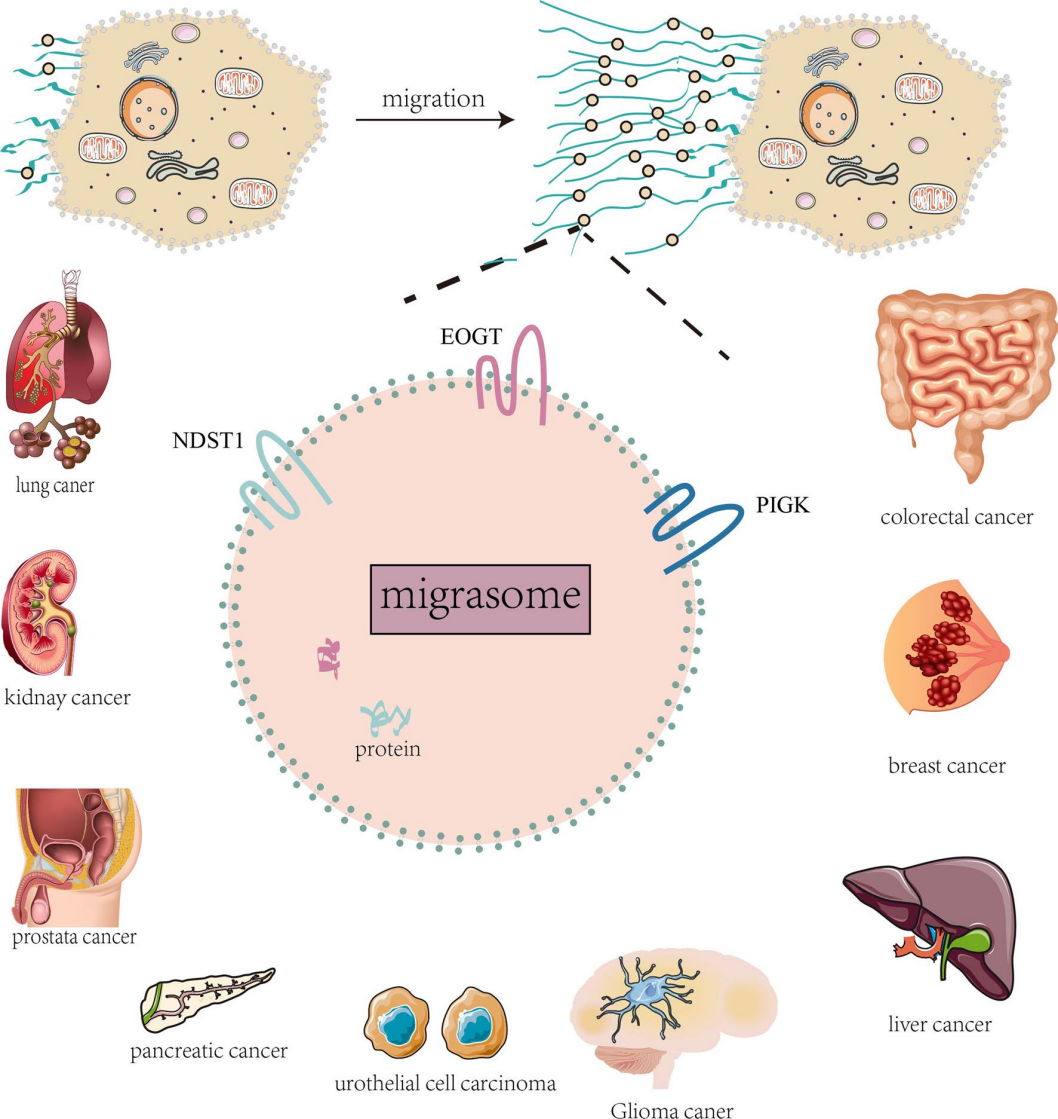
**Formation of migrasome and its specific marker proteins associated with cancers**. Cell migration, as an adaptive process, is crucial to normal physiological processes and pathology of multicellular animals. It is also a hallmark of cancer malignancy. After cell migration, migrasomes are formed on the tips or intersections of the RFs (retraction fibers).Migrasomes are newly discovered vesicular cellular organelle with diameters of 0.5–3 µm produced by migrating cells and depending on cell migration, form on the RFs trailing behind migrating cells. Specific marker proteins of migrasome are closely associated with various cancers such as lung cancer, kidney cancer, prostate cancer, pancreatic cancer, urothelial cell carcinoma, glioma cancer, liver cancer, breast cancer, and colorectal cancer

Migrasomes depend on cell migration[14], form on the RFs trailing behind migrating cells[15], and first described by Ma and colleagues[7] (Fig. 1). However, the specific mechanism of migrasome formation is still less clear. To elucidate the molecular mechanism underlying migrasome biogenesis in detail, many scientists have made great efforts in the study of migrasomes.

The production of migrasomes may be monitored by proteins involved in cell-extracellular microenvironment interactions. Migrasomes form on the RFs trailing behind migrating cells[15]. And RFs must adhere to the extracellular matrix (ECM). The integrins in the cells are enriched in the focal adhesions (FAs) that connect the cells and the extracellular matrix (ECM). Of note, it has been clarified that integrin α5β1 was enriched on the bottom side of the migrasome. Based on the above speculation, the location of migrasomes formation can be roughly predicted by the location of integrin enrichment. In the depth of their research, they suggested that migrasomes are not FAs. Subsequently, they concluded that migrasomes formation depends on the proper adhesion of integrins to specific ECM partners[16]. Integrins are gathered into puncta on RFs before migrasome formation, and that integrins-ECM interactions are necessary to establish the adhesion sites along RFs[17]. In 2019, Huang et al.[18] used live-cell experiments, a theoretical model, and a membrane-stiffening effect to confirm that tetraester-rich and cholesterol-rich membrane microdomains assemble into large micron-scale domains that then expand into migrasomes. Ultimately, they concluded that micrometer-scale membrane microdomains, assembled from membrane microdomains rich in tetraspanins and cholesterol, promote the formation of migrasome formation. And the presence of migrasome that must produce such micrometer-scale membrane microdomains[18]. The reason why the tetraspanin-enriched macrodomains drive the formation of spherical structures on thin film tether chains was revealed through the membrane stiffening model (a theoretical model), which predicts that the high membrane rigidity of tetraspanin-enriched macrodomains promotes the expansion of the migrasome induced by retraction fibers[19]. Additionally, fibronectin is an important factor regulating migrasome formation. Lu et al.[14] found that ROCK1 can regulate cell adhesion to fibronectin and the ROCK1 inhibitor SAR407899 can inhibit migrasome biogenesis in vivo in a zebrafish model system. So they concluded that ROCK1 can serve as a regulator of migrasome formation. In 2021, Saito et al.[20] found that migrasomes can form on peptide-modified substrates and peptide-modified substrate contributes to cell migration and migrasome formation. Specifically, peptide scaffolds on substrates have been shown to be critically relevant to cellular function. Peptide scaffolds include cell penetrating, virus fusion, and integrin-binding peptides which enable the formation of migrasome-like vesicles. Through structural and functional analyses, they confirmed that migrasomes formed on these peptide-modified substrates. The peptide interface comprising cell-penetrating peptides (pVEC and R9) and virus fusion peptide (SIV) have superior properties for enabling the migrasome formation than fibronectin protein, integrin-binding peptide (RGD), or bare substrate.

The contents of the migrasome are variable and not fixed that could contain Chemokines[21], cytokines[21], growth factors[22], mRNA[23], some proteins[16], damaged mitochondria[24], and more. The contents of the migrasome may determine its corresponding biological function. For example, chemokines or cytokines promote a correct zebrafish embryogenesis[24]. mRNAs and proteins participate in the cell proliferation modulation[13]. Removal of damaged mitochondria ensures mitochondrial homeostasis within the cell[25].

## The biological functions of migrasomes

**Fig. 2:**
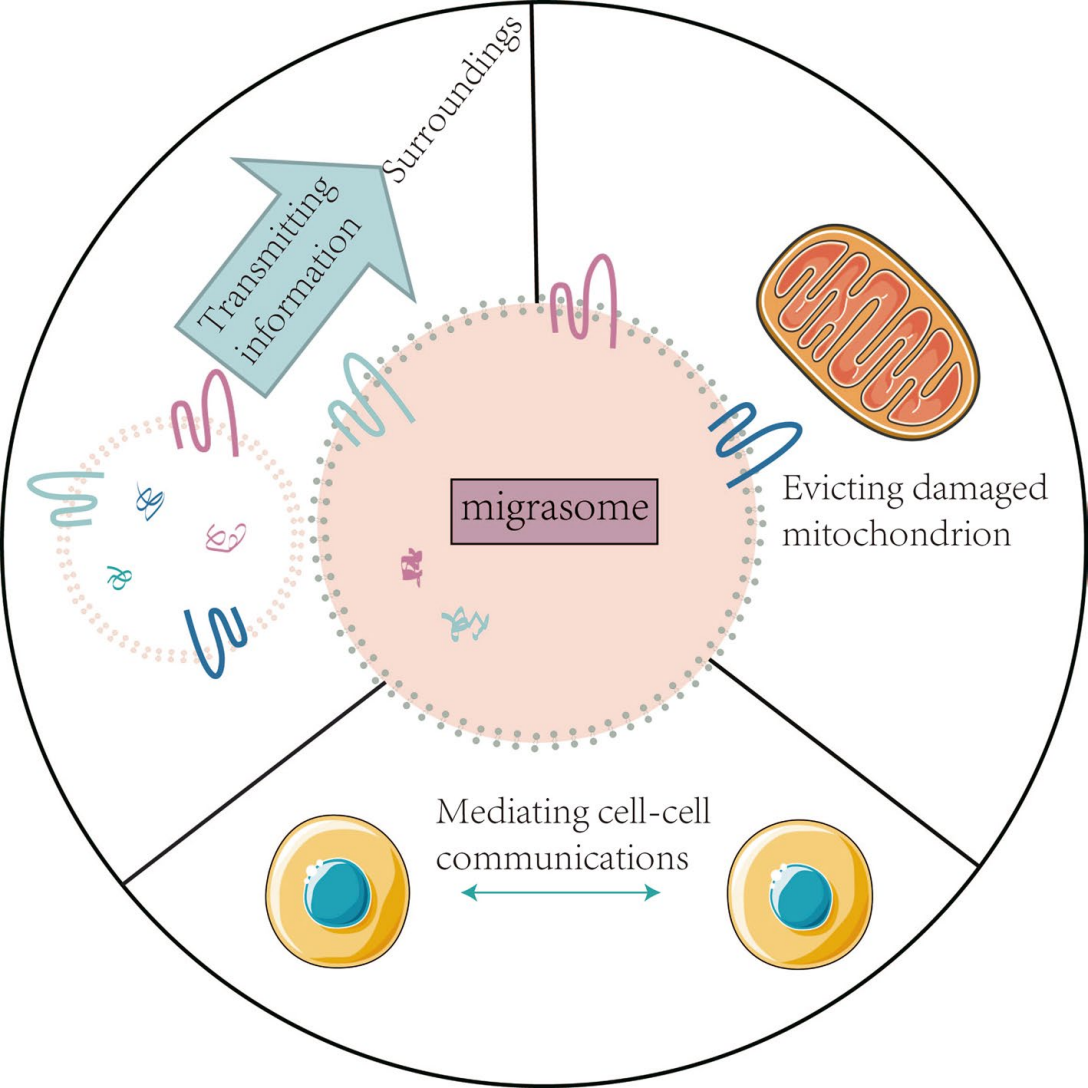
**The biological function of migrasome**. Migrasomes mediate multi-intercellular communication, transmit to a spatially defined location that signals to the surrounding cells in the microenvironment, participate in mitochondrial quality control during mitosis, maintain mitochondrial homeostasis, and mediate the lateral or horizontal transfer of RNAs and proteins

Cell migration prompted disruption of connections between cells and the RFs and subsequent RFs disintegration, eventually leading to complete detachment of the migrasome from the cell. Currently, the biological functions of migrasomes are mostly reported to mediate multi-intercellular communication[24], transmit to a spatially defined location that signals to the surrounding cells in the microenvironment[25], participate in mitochondrial quality control during mitosis and maintain mitochondrial homeostasis[19] (Fig. 2).

The migrasomes contain some contents of the cell, and it eventually breaks up as the cell migration movement ends, and its content is released outside the cell. Therefore, migrasomes are proposed as a mechanism mediating multi-intercellular communication and involvement in the regulation of physiological and pathological processes. Additionally, migrasomes can also serve as information packets that can be transmitted to a spatially defined location that signals to the surrounding cells in the microenvironment. For example, Jiang et al.[24] found that migrasomes contribute to the formation of organs via serving as chemoattractants on a cavity underneath the embryonic shield, which showed that migrasomes act as signaling organelles providing specific biochemical information to coordinate the morphogenesis of organ. Migrasomes can expel damaged organelles from cells and have been investigated as a waste disposal mechanism. For example, migrasome participate in mitochondrial quality control during mitosis and maintain mitochondrial homeostasis[25]. It has also been shown that migrasomes are partly involved in mediating the lateral or horizontal transfer RNAs and proteins[19].

Overall, the biological function of migrasomes is mainly to mediate cell-to-cell communication and thus to act as an information transmitter. However, in the tumor microenvironment, cancer cell-cancer cell, cancer cell-non-cancer cell communication, and related cell migration behavior, is shown to play a crucial role in the development of cancer[26]. From above, we can confidently associate the migrasome with cancer.

## Identification of migrasomes and migrasomes in disease

### Identification of migrasomes

Chen et al.[17] described detailed methods for visualizing migrasomes in cells either by fluorescence microscopy or electron microscopy. Specifically, using TSPAN4-GFP as a migrasome marker, it is possible to label migrasomes and observe their structure by confocal microscopy during migration of living cells[17]. Transmission electron microscopy (TEM) can clearly demonstrate the ultrastructure of migrasomes in various types of cells. However, on this basis, they immediately discovered the great drawbacks of this method (using a fluorescently tagged marker protein for detection of an organelle). This method has two main limitations: First, it is time consuming. Second, the overexpressed marker protein may change the biogenesis of migrasomes, thus causing artifacts. Immediately following this, in 2019, they also found a probe-WGA that facilitates the rapid detection of migrasomes in both fixed and living cells[27]. They found that the WGA signal on migrasomes was much higher than the WGA signal on retraction fibers by florescence intensity analysis, indicating that WGA prefers to bind to migrasomes[27]. Keeping WGA in the culture medium during imaging gave a reasonable signal-to-noise ratio and enable to monitor migrasomes formation for a much longer periods of time[27]. Moreover, long-term exposure to WGA had only slight effects on the migrasomes biogenesis and cell migration, and the formation of migrasomes was not affected by the presence of WGA[27].

## Migrasomes in disease

Studies on the role of migrasomes in disease are only beginning and in very small numbers. It has been found that migrasomes affect the cytoplasmic release of neurons around the brain of stroke patients as well as their involvement in post-stroke regulation[28]. Their studies demonstrated that migrasomes play a crucial role in sodium chloride-driven acute ischemic stroke. In 2020, Liu et al.[29] found that injured podocytes produced more migrasomes than healthy podocytes, from which they proposed that urinary podocyte migrasomes are promise as a diagnostic marker for early podocyte injury in diabetic nephropathy patients.

It is also found that migrasomes play a biological role in organism development, innate immunity, COVID-19, cardiovascular and cerebrovascular diseases, kidney diseases and cancer biology[30]. Macrophages are capable of generating migrasomes. The number of migrasomes were significantly reduced in the bone marrow-derived macrophages (BMDMs) derived from TSPAN9-/- mice compared with wild-type (WT) mice[25]. Most of the migrasomes in the mouse circulation originate from neutrophils, and these migrasomes can adhere to the mouse blood vessels for long periods of time[25, 31].

Hyperactive platelets in patients with coronavirus disease 2019 (COVID-19) presenting as hypercoagulation and thrombosis can lead to the release of tissue factors from monocytes and eventually to serious thromboinflammation[32, 33]. It is noteworthy that migrasomes can control the formation of thrombotic inflammation under this mechanism[34].

There is little direct literature evidence for the role of migrasomes in cancer, but it is mainly described around non-specific marker of migrasome or migrasome-mediated protein in cancer. It has been speculated that migrasomes may also be present in multiple cancer cell types based on its markers[15]. Migrasomes might be a particularly attractive type of signalling vesicles in atherosclerosis due to the high rate of immune cell migration[35]. Migrasome-mediated transfer of Pten mRNA and Pten protein can inhibit the proliferation of Pten-deficient breast cancer cell line (MDA-MD-468) [23]. Atherosclerosis can impact cancer progression due to the cholesterol and calcium metabolism[36]. Tetraspanin 4 (TSPAN4) was highly expressed in atherosclerosis and pan-cancer, which was associated with the progression and immune cell infiltration of the tumor, especially in Glioblastoma multiforme (GBM)[37]. And TSPAN4 serves as a required protein for migrasome formation[24]. Therefore, it can be speculated from above that the investigation of TSPAN4 functions for GBM treatment may help understand migrasomes in cancer. In addition, the TSPAN4 and migrasomes in macrophages associated with myocardial infarction and pan-cancer progression may be key to the treatment of patients with cardiovascular disease[38]. The investigators identified migrasomes without TSPAN overexpression in neural crest cells (NCCs)[39]. The downregulated TSPAN4 was able to inhibit gastric cancer tumor formation[40]. High expression of TSPAN4 in lung adenocarcinoma (LUAD) promotes its metastasis and progression[41]. To sum up, we can guess that TSPAN4 of migrasomes may be a highly promising therapeutic target for some cancers. The interaction between Multipotent mesenchymal stromal cells (MSCs) and cancer cells can enhance the cancer and metastatic potential[42]. Deniz et al.[43]demonstrated that plastic-adherent MSCs isolated from human bone marrow generate migrasomes via detecting the MSC markers (CD44, CD73, CD90, CD105 and CD166) which are present on the migrasome network.

## The differences between migrasomes and exosomes in diameter, formation, final destination, specific recognation function, and function

As for the relationship between exosomes and migrasomes, we have explored their differences in terms of their diameter, essence, formation[19, 44], their final destination, function, and whether they have a specific recognition function as for the relationship between exosomes and migrasomes (Table 1).

**Table 1:** The differences between migrasomes and exosomes in diameter, essence, formation, final destination, specific recognition functions, and function

	Exosomes	Migrasomes
Diameter	0.03–0.15 μm[45]	0.5–3 μm[14]
Essence	Extracellular vesicles (EVs)[13]	Organelle[14]
Formation	The plasma membrane is invaginated to form an Early sorting Endosome, which is modified to become a Late sorting Endosome The second invagination that occurs in Late sorting Endosome creates the Intraluminal vesicles. A collection of many Intraluminal vesicles becomes Multivesicular Body. Then Multivesicular Body binds to the plasma membrane and secretes exosomes by exocytosis [44]	First, a bright spot was formed on the retraction fibers, and then the TSPAN4 protein gradually enriched on migrasomes. It was a small spot at first, and then gradually grew into a vacuolar structure [19]
Final destination	Usually transported to distant cells [48]	It is taken up by surrounding cells, and because the location of the migrasomes is usually fixed, the cellular ingestion of the migrasomes results in good positioning for subsequent communication with other organelles or cells [15]
Specificity	Binds specifically to downstream targets [48]	Still uncertain
Function	Traditionally, exosomes act as cell scavengers, responsible for removing the waste generated from cell physiological activities to maintain normal physiological homeostasis[44]. Recent boom studies in exosomes have focused on exosome-mediated signaling and molecular transfer, which suggest that exosomes play a vital role in the modulation of intercellular communication[49]	Mediate multi-intercellular communication, transmit to a spatially defined location that signals to the surrounding cells in the microenvironment[24], participate in mitochondrial quality control during mitosis and maintain mitochondrial homeostasis[25]. Mediate the lateral or horizontal transfer of RNAs and proteins[19]

Exosomes are assigned to the smallest Extracellular vesicle (EV) sub-population. On the one hand, exosomes are EVs of 0.03–0.15 μm[45] and are created by budding at both plasma and endosome membranes. On the other hand, exosomes are components and remodelers of the extracellular matrix[46, 47]. However, migrasomes (0.5-3 μm) are novel organelles[14] belonging to the large EVs category[13]. They have been described as pomegranate-like structures, large vesicles encapsulating numerous smaller vesicles ranging 0.05–0.1 μm. The formation of migrasomes seems to be much simpler: First, a bright spot was formed on the retraction fibers, and then the TSPAN4 protein gradually enriched on migrasomes. It was a small spot at first, and then gradually grew into a vacuolar structure[19]. After release, exosomes are usually transported to distant cells [48], but migrasomes are usually taken up by surrounding cells. Because the location of the migrasomes is usually fixed, the cellular ingestion of the migrasomes results in good positioning for subsequent communication with other organelles or cells [15]. Exosomes can bind specifically to downstream targets [48]. However, the specific binding relationship of the migrasome is not yet known, but we may be able to make appropriate inferences by its specific markers. Traditionally, exosomes act as cell scavengers, responsible for removing the waste generated from cell physiological activities to maintain normal physiological homeostasis[44]. Recent boom studies in exosomes have focused on exosome-mediated signaling and molecular transfer, which suggest that exosomes play a vital role in the modulation of intercellular communication[49]. The biological function of migrasomes is similar to the function of exosomes, that is, both removing the garbage from the cells. In addition, migrasomes can also mediate multi-intercellular communication, transmit to a spatially defined location that signals to the surrounding cells in the microenvironment[24], participate in mitochondrial quality control during mitosis, maintain mitochondrial homeostasis[25], and mediate the lateral or horizontal transfer of RNAs and proteins[19].

There are very few available methods to detect migrasomes. However, exosomes have been studied for a relatively long time, so we have summarized the separation and analysis methods of exosomes and their advantages and disadvantages, hoping to have some enlightenment for the exploration of the analysis method of migrasomes in the later stage. Currently, exosome analysis methods are the following: Transmission electron microscope[50], Resistive Pulse Sensing[51], Atomic force microscope measurements[52], and flow cytometer[53]. The transmission electron microscope can retain the morphology of the exosome biological structure and low cost, but is insensitive to macromolecules such as proteins and polysaccharides. From this, we can speculate that this method may be insensitive to the signature proteins on the migrasome, thereby inefficiently detecting the presence of the migrasome[50]. Homoplastically, resistive pulse sensing can measure individual size and distribution of exosomes, but blockage easily occurs when particles are too large. We know that the size of exosomes is ~ 30 nm to ~ 200 nm[45] and the size of migrasomes is 500 nm to ~ 3000 nm[13], so this method is more prone to blockage when used for migrasomes detection[51]. Flow cytometer can preserve the morphology of exosome biological structures, but sample fixation and dehydration can affect the size and morphology[53]. Atomic force microscope (AFM) measurements may be the best choice because it has higher sensitivity, but is more expensive[52].

The isolation and purification of exosomes seriously limit the clinical application of exosomes due to the cumbersome methods, time-consuming, low yield, and low purity. Given that migrasomes are extremely similar to exosomes, we can imagine that the isolation and purification process of migrasomes would also encounter these similar difficulties. Ultracentrifugation is the current mainstream exosome isolation method[54], but it still has problems such as low yield, poor integrity of exosomes, and long time. In addition, for some specific cases, there are methods such as polyethylene glycol (PEG)-based precipitation[55], phosphatidylserine affinity capture, size-exclusion chromatography[56] and membrane affinity[57], and other separation methods[58], but from the results, they have little effect. The asymmetric flow field-flow fractionation method can screen and isolate exosomes with high resolution[59], but this method requires high concentrations of exosomes and cannot be widely used in clinical research. Recently, in the study of Yu et al.[60], an efficient method for the detection of exosomes by an exosome detection method via the ultra fast-isolation system EXODUS was described, which compared with ultracentrifugation, not only the yield and purity are improved, but the time required for separation and purification is also shortened. So ultrafast-isolation system EXODUS is likely to be applied to the migrasomes. Overall, the above detection and separation analysis methods of exosomes and their advantages and disadvantages can provide some enlightenment for the exploration of later migrasomes analysis methods.

## Specific markers of migrasomes( not present in exosomes) in cancer

At first sight, migrasomes are very similar to exosomes, and they are all extracellular membrane-bound vesicular structures[18]. There is still a big difference between the two, including the process of release, size, detection method, and markers[18]. Multiple lines of evidence demonstrated that exosomes are closely related to human cancer[61]. For example, exosomes are associated with prostate cancer (PCA)[62], hepatocellular carcinoma (HCC)[63], renal cell carcinoma (RCC)[64], pancreatic cancer[65] and glioblastoma (GBM)[66] etc. Additionally, exosomes play a pivotal role in the drug resistance of cancer cells[66]. However, few articles have been reported on the potential role of migrasomes and migracytosis in cancer, especially in tumor cell migration. Zhao et al.[67] successfully identified marker proteins (not present in exosomes) NDST1, EOGT, PIGK, and CPQ for migrasomes. Understanding the function of these marker proteins in cancer (especially in cancer cell migration) is likely to help us to investigate, understand and elucidate the possible biological functions of migrasomes and migracytosis and their potential implications in human cancer. Clinical treatment of migrasomes of cancer using a biomarker-driven approach should be considered in the future. Since there is no literature reporting CPQ on cancer, we only reviewed the relevant role of the first three markers in cancer (Table 2 and Fig. 1). A detailed understanding of the migrasome-specific markers could speculate that migrasomes would also most likely influence the biogenesis of these cancers associated with migrasome-specific markers by mediating migrasome-specific markers.

**Table 2:** Specific markers of migrasomes in cancer (not present in exosomes)

Marker proteins	Cancer	Functions	Reference
NDST1	clear cell renal cell carcinoma	prognostic signatures	[84]
NDST1		tumor angiogenesis	[83]
NDST1	lung cancer	target DC glycan sulfation	[82]
NDST1	breast cancer	cell proliferation and migration	[85]
NDST1	renal carcinoma	cell invasion, metastasis and migration	[87]
NDST1	breast cancer	chemoresistance	[86]
NDST1	gastric carcinoma	cell growth	[88]
NDST1	primary glioblastoma	anti-oncogene	[89]
NDST1	early KRAS-mutant lung neoplasia	acquired anti-tumor T cell immune	[81]
NDST1	prostate cancer	cell proliferation and migration	[91]
EOGT	pancreatic cancer	cell proliferation and migration	[99]
EOGT	hepatocellular Carcinoma (HCC)	unfavorable prognostic indicator	[100]
EOGT	pancreatic cancer	cell proliferation, migration, invasion and pancreatic cancer onset and progression	[98]
PIGK	colorectal cancer (CRC), HCC and urothelial cell carcinoma (UCC)		[105]

## NDST1 in cancer

NDST1 is HS (Heparan sulfate) metabolism-involved gene, which can reduce heparan sulfate sulfation[68]. Atienza et al.[69] developed a fluorometric coupled enzyme assay (radioactive labeling assays) to determine the activity of NDST that is utilized to assess potential enzyme inhibitors for drug development. NDST1 is found overexpressed in mature oligodendroglia (OLG) bordering the lesion and the number of NDST1-expressing oligodendroglia is inversely correlated with lesion size[70]. Deleting NDST1 significantly caused a decrease in the binding affinity of both vaccinia and myxoma viruses to the cell surface[71]. Selective Deletion of Heparan Sulfotransferase Enzyme, NDST1, in Donor Endothelial and Myeloid Precursor Cells Significantly Decreases Acute Allograft Rejection[72]. TM9SF2, essential for CHIKV infection of HAP1 cells, has been found to be involved in the N-sulfation of heparan sulfate via ensuring NDST1 activity[73]. It has been found that NDST1 is related to Pathologic Lymphangiogenesis[74] and lymph node metastasis[75]. Additionally, NDST1 is closely implicated in human disease, including Atherosclerosis[76], Holoprosencephaly[77], Congenital diaphragmatic hernia (CDH)[78], Osteoarthritis (OA)[79], allergic airway inflammation (AAI)[80]. Notably, much of the recent NDST1 research is focused on human cancer. NDST1 can influence tumor T cell immune mechanisms[81], tumor Growth[82], tumor angiogenesis[83], etc.

It is well known that lymph node metastasis is a key event in tumor progression. However, heparan sulfate may play a crucial role in lymphatic metastasis as a mediator of chemokine action. Interestingly, NDST1 is involved in the ascending heparan sulfate chains. So it is not surprising to link NDST1 to tumor development. Zeng et al.[84]found that NDST1 is a promising prognostic marker in clear cell renal cell carcinoma. Yin et al.[75] altered lymph node metastasis in tumor-bearing gene-targeted mice via downregulating the expression of NDST1. They reported that the mutations in the HS gene in lymphatic endothelial cells inhibit the chemokine-dependent migration of tumor cells to endothelial cells. Deletion of NDST1 in the lymphatic endothelium in vivo alters lymph node colonization by the tumor. Alterations in the endothelial cell sulfate liver-associated gene NDST1 can selectively inhibit tumor angiogenesis without affecting physiological angiogenesis[83]. It has been found that tumor-associated dendritic cells (DCs) play a pivotal role in cancer cell growth. NDST1 mutations in DCs can affect the activation of major signaling pathways required for the migration of lymphatic-driven DCs and the migration of chemokine (CCL21)-dependent DCs. Mutation/Deletion of NDST1 can target DC glycan sulfation, increasing the maturation of DC and inhibiting the process that trafficking of DCs to draining lymph nodes, thereby reducing the lung cancer tumor volume in mice[82].

In human breast cancer, HS agonist heparin increased NDST1 expression. Rachel et al.[85] found that the above responses involved in HS are closely related to the Wnt signaling, and ultimately play an effect in promoting breast cancer cell proliferation and migration. Interestingly, NDST1 is also related to chemoresistance in human breast cancer. Hypermethylation of miR-149 downregulates its an expression and increase NDST1 expression, thereby leading to chemoresistance for breast cancer[86]. In addition, knocking down NDST1 contributes to suppressing HS production, which results in suppressed renal carcinoma cell invasion, metastasis, and migration[87]. MiR-191 targets NDST1 and promotes gastric cancer cell growth in human gastric carcinoma cell line MGC803[88]. It plays an oncogenic role in primary glioblastoma via targeting NDST1[89]. Kim et al.[81] found that loss of function of the glycan sulfating enzyme NDST1 targeted to antigen-presenting cells (APCs) may enhance acquired anti-tumor T cell immunity, inhibiting early KRAS-mutant lung neoplasia. Heparan sulfate proteoglycans have been investigated as key components of the cell microenvironment and have been demonstrated to be related to cell–cell interactions, migration, and signaling. HS metabolism-involved genes, such as NDST1, were studied to uncover cancer-related changes in the transcriptional pattern of the HS biosynthetic system. It has been found that HS-biosynthetic machinery in cancer cells in vitro or vivo have e cell type-specific changes or tissue-specific changes[90]. Anastasia et al.[91] found that changes in the expression levels of all HS metabolism-involved genes (EXT1, EXT2, NDST1, NDST2, GLCE, 3OST1/HS3ST1, SULF1, SULF2, HPSE) can inhibit the HS-metabolic system in prostate cancer, thereby influencing tumor cell proliferation and migration. These findings sustain NDST1 as the function of the tumor suppressor gene. From this, we can also make some appropriate suggestions to speculate that the migrasome may play a role in regulating the biological process of related cancers above via mediating NDST1.

## EOGT in cancer

EOGT, an endoplasmic reticulum (ER)-resident protein, regulates Notch signaling by modifying EGF repeats of Notch receptors and thereby mediates glycosylation[92]. EOGT is closely linked to human diseases. It has been found that EOGT is related to vascular development in mammals[93]. Shaheen et al.[94] found that EOGT is one of the causative genes of a congenital disease, Adams-Oliver syndrome (AOS). Additionally, Sakaidani et al.[95] reported that EOGT served as O-linked-N-acetylglucosamine (O-GlcNAc) transferase, which is responsible for extracellular O-GlcNAcylation and involved in the etiology of diabetes and neurodegeneration. Muter et al.[96] revealed that knockdown of EOGT perturbed a network of decidual genes involved in multiple cellular functions, which are involved in energy homeostasis and glucose and fatty acid metabolism. However, obesity impacts the EOGT-adropin axis in decidual cells, which points toward a mechanistic link between metabolic disorders and adverse pregnancy outcomes.

Activation of the Notch signaling has been demonstrated to regulate the development of pancreatic cancer[97]. Yang et al.[98] demonstrated that SHCBP1 (Shc SH2-domain binding protein 1) interacts with EOGT, facilitating O-GlcNAcylation of NOTCH1, thereby promoting pancreatic cancer cell proliferation, migration, invasion, and pancreatic cancer onset and progression. Bioinformatics and functional analyses of Barua et al.[99] showed that EOGT as a Notch-modifying glycosyltransferase regulated the proliferation and migration of pancreatic cancer cells and overall survival in pancreatic ductal adenocarcinoma (PDAC) patients. EOGT in Hepatocellular Carcinoma (HCC) correlated with immune infiltration whose expression was significantly higher than that in normal tissues, and it is associated with advanced tumor stage and poor overall survival. Thus, EOGT was considered as a significant poor prognostic indicator for HCC patients[100]. Generally, EOGT has great promise as a new biomarker, which reflects the progression of pancreatic cancer and HCC. We might be able to start with EOGT and go deeper into the migrasomes produced by these two cancer cells. By controlling the formation of their migrasomes, perhaps providing an emerging research direction for the treatment of these two cancers.

## PIGK in cancer

PIGK (GPI8), one of the five subunits that make up Glycosylphosphatidylinositol-transamidase (GPI-TA) complex and a quality control factor in the GPIT complex, plays an essential role in protein-GPI anchoring[101].

It has been proposed that PIGK was correlated with human diseases. Okamura et al.[102] firstly demonstrated that PIGK might affect tyrosinase activity in human melanocytes. Nguyen et al.[103] found that Bi-allelic Variants in PIGK cause Neurodevelopmental Syndrome with Hypotonia, Cerebellar Atrophy, and Epilepsy. Additionally, loss of PIGK function causes severe infantile encephalopathy and extensive neuronal apoptosis[104]. However, the role of PIGK in tumorigenesis remains largely unknown. Nagpal et al.[105] found that PIGK presents a low expression condition in a variety of cancers, such as colorectal cancer (CRC), HCC, and urothelial cell carcinoma (UCC). They hypothesized that the mutations in the coding region of the PIGK gene may lead to altered PIGK expression. In 2012, research demonstrated, for the first time, that SNP1048575 was related to low PIGK expression in CRC/ patients and a possible association between altered PIGK expression and disease susceptibility by direct sequencing and immunohistochemistry[106]. From what has been discussed above, for CRC/HCC/UCC, PIGK is a promising potential therapeutic target. The investigation of PIGK functions may help understand migrasomes in cancer and provide novel targets for treatment.

## Perspective

**Fig. 3:**
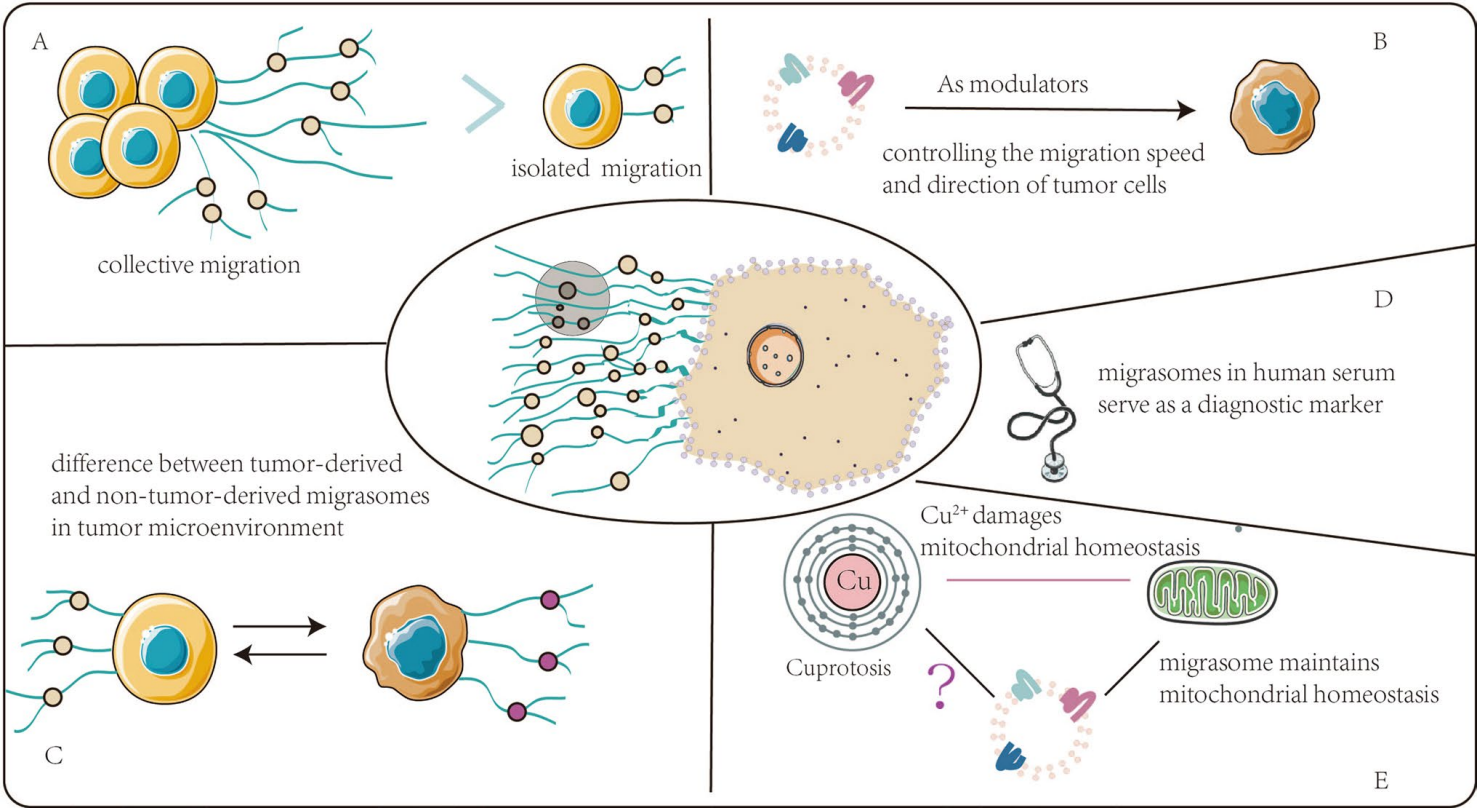
**Perspective.** (A) Collective migration is more efficient than single cell migration, probably because migrasomes serve as signaling communicators. (B) Migrasomes may serve as modulators in cell migration and control the migration speed and direction of tumor cells. (C) Tumor-derived and non-tumor-derived migrasomes in the tumor microenvironment may play different functions. (D) The migrasomes in human serum may be used as biomarkers for tumor prognosis like exosomes. (E) Cuprotosis damages the mitochondria, but migrasome clears the damaged mitochondria, whether there is an upstream and downstream mechanism between Cuprotosis and migrasome?

In this section, we developed a series of bold conjectures for future applications of migrasomes (Fig. 3).

Cell migration can be divided into two categories: collective cell migration and isolated cell migration. Single cells have the advantage of a high instantaneous speed, but they have the disadvantages of continuously migrating less and frequently changing their direction. Collective cell migration plays a key role in cancer metastasis[107]. Collectively migrating cells are more efficient than migrating separate cells, suggesting that cells interact during collective migration[108]. During collective migration, multiple cells migrate in the same direction at a similar speed, and they will regulate their surrounding environment during the migration, pulling those cells that were originally stationary or migrated in different directions to undergo the collective mass migration at the same speed and direction. It can therefore be concluded that the collective cell migration is indeed more efficient than the migration of the isolated cells. From this, we propose the following scenario: Collective migration is more efficient because more migrasomes arise as signalators.

The leader cells play a key role in promoting collective movement[109]. Uncovering the mechanisms by which these leading cells emerge and the molecular properties of these cells will potentially help us to better understand how migrating cells arise from nonmigrating tissues in cancer. However, a leader has a follower. The followers proposed by the scientists may be active participants in controlling the speed and direction of migration[110]. All kinds of information reveal that followers can convey through direct contact, soluble factor exchange, and changing the microenvironment, which suggests that leader cells in migrating cells are largely influenced by followers, and that understanding the intercellular signals for intracellular exchange may be a new revelation for promoting or inhibiting collective migration[108]. Since that intercellular communication is also involved, we conjecture that can migrasomes act as such followers and thus participate in controlling the migration speed and direction of tumor cells.

Cell migration, a hallmark of cancer malignancy, plays a critical role in cancers. However, migrasomes are produced by migrating cells and depending on cell migration. It seems likely that migrasomes also play an extremely important role in the biological processes of migrating cells, including some pathological conditions such as tumor metastasis. It is reasonable to speculate that migration-dependent migrasomes and migracytosis might be novel players in mediating cancer development. However, the origin of migrasomes, targets, as well as biological functional effects in human cancers remains to be explored. In particular, we can venture to question, can the migrasome in human serum[67]serve as a diagnostic marker in certain cancers? Since exosomes in the tumor microenvironment can be divided into tumor-derived and non-tumor-derived exosomes, is thus also suitable for migrasomes? Tumor-derived or non-tumor-derived exosomal noncoding RNAs regulate the development of human cancer through different signaling pathways in the tumor microenvironment[49]. What's the difference between tumor-derived and non-tumor-derived migrasomes in tumor microenvironment? Additionally, in 2022, Tsvetkov et al.[111] showed that copper mediates copper-dependent death by directly binding to the lipoylated components in the tricarboxylic acid (TCA) cycle. They found that treatment of mitochondrial function has a very clear effect on the sensitivity of copper ionophore, copper-binding small molecule that shuttle copper into the cell and useful tool to study copper toxicity. Notably, migrasome mediated mitochondrial quality control to maintain mitochondrial homeostasis[25]. What are the effects of migrasomes on copper-dependent death in tumor cells?

According to the main questions about migrasomes raised by Yu et al.[19] and the content of our review, we tried to elaborate the possible related diseases and mechanisms of migrasomes from the perspective of migrasome-specific markers, perhaps suggesting some new insights into the biological role of migrasomes in pathological situations. However, similar to their study, we do not know the dynamics of migrasomes in vivo, and perhaps more in vivo studies of migrasomes in pathological and physiological situations will be needed to better understand their biological roles. The answers to these questions require more studies combining various cell experiments with animal models.

## Data Availability

The datasets used or analysed during the current study are available from the corresponding author on reasonable request.
